# Microwave Imaging Sensor Using Compact Metamaterial UWB Antenna with a High Correlation Factor 

**DOI:** 10.3390/ma8084631

**Published:** 2015-07-23

**Authors:** Md. Moinul Islam, Mohammad Tariqul Islam, Mohammad Rashed Iqbal Faruque, Md. Samsuzzaman, Norbahiah Misran, Haslina Arshad

**Affiliations:** 1 Space Science Centre (ANGKASA), Research Centre Building, Universiti Kebangsaan Malaysia (UKM), Bangi, Selangor D. E. 43600, Malaysia; E-Mails: mmoiislam@siswa.ukm.edu.my (M.M.I.); rashed@ukm.edu.my (M.R.I.F.); 2 Department of Electrical, Electronic and Systems Engineering, Faculty of Engineering and Built Environment, Universiti Kebangsaan Malaysia (UKM), Bangi, Selangor D. E. 43600, Malaysia; E-Mails: sobuzcse@eng.ukm.my (M.S.); bahiah@eng.ukm.my (N.M.); 3 Center for Artificial Intelligence Technology, Faculty of Information Science & Technology, Universiti Kebangsaan Malaysia (UKM), Bangi, Selangor D. E. 43600, Malaysia; E-Mail: haslinarshad@ukm.edu.my

**Keywords:** breast cancer, correlation factor, metamaterial, ultra-wideband

## Abstract

The design of a compact metamaterial ultra-wideband (UWB) antenna with a goal towards application in microwave imaging systems for detecting unwanted cells in human tissue, such as in cases of breast cancer, heart failure and brain stroke detection is proposed. This proposed UWB antenna is made of four metamaterial unit cells, where each cell is an integration of a modified split ring resonator (SRR), capacitive loaded strip (CLS) and wire, to attain a design layout that simultaneously exhibits both a negative magnetic permeability and a negative electrical permittivity. This design results in an astonishing negative refractive index that enables amplification of the radiated power of this reported antenna, and therefore, high antenna performance. A low-cost FR4 substrate material is used to design and print this reported antenna, and has the following characteristics: thickness of 1.6 mm, relative permeability of one, relative permittivity of 4.60 and loss tangent of 0.02. The overall antenna size is 19.36 mm × 27.72 mm × 1.6 mm where the electrical dimension is 0.20 λ × 0.28 λ × 0.016 λ at the 3.05 GHz lower frequency band. Voltage Standing Wave Ratio (VSWR) measurements have illustrated that this antenna exhibits an impedance bandwidth from 3.05 GHz to more than 15 GHz for VSWR < 2 with an average gain of 4.38 dBi throughout the operating frequency band. The simulations (both HFSS and computer simulation technology (CST)) and the measurements are in high agreement. A high correlation factor and the capability of detecting tumour simulants confirm that this reported UWB antenna can be used as an imaging sensor.

## 1. Introduction

The field of electromagnetic waves and antennas has attracted increasing interest for the medical application of microwave systems. Microwave imaging is an example of using such a system for detecting breast cancer [1,2,3,4,5]. A microwave imaging sensor is used to identify the contrast between the electrical properties of human tissues. Power is radiated through an antenna in a microwave imaging system and one or more antennas receive the scattered power. To detect unwanted cells (targets), the scattered signals are then resolved. The ultra-wideband (UWB) pulse provides stable penetration and resolution characteristics. These typical microwave imaging systems have been suggested for detecting hidden breast cells [6,7,8]. 

The design of metamaterial UWB antennas raises significant challenges to implement all the categories of microwave imaging. Different types of antennas are proposed for microwave imaging (mainly tissue sensing) applications such as a cross-Vivaldi antenna [9], a planar dark eyes antenna [10], a planar monopole [11], a ridged pyramidal horn [12], a slot antenna [13], a TEM horn antenna [4] and the Fourtear antenna [14]. A metamaterial which is not available in nature, holds an artificial electromagnetic structure with negative permittivity and /negative permeability over a specific frequency range. A new period for metamaterials in microwave imaging applications is created due to the great potential use of metamaterials in effective microwave devices development such as antennas. 

In 1968, Veselago reported the theoretical prediction of an engineered material showing negative permittivity and negative permeability simultaneously [15]. In 1999, Pendry demonstrated metamaterials based on the split ring resonator (SRR) [16] and ultimately, in 2000, Smith effectively demonstrated and validated the metamaterial (negative µ and ɛ) concept [17]. Various metamaterials (left-handed) have been described using different shapes, such as split ring resonator (SRRs) [18], multiple SRRs [19], fishnet structures [20], spiral SRRs [21], double-sided SRRs [22], layouts of transmission line [23], H-shaped pairs periodic arrays [24], double-bowknot shaped resonators [25], SRR pairs [26], cut wire pairs [27], complementary resonator of electric field-coupled [28] and broad side coupled SRRs [29]. The area of metamaterials research is able to enhance a variety of technologies. However, due to the limited frequency band, the range and spectrum of their applications are restricted. It is difficult to fabricate and use these materials in antenna design. Therefore, the fields of metamaterial application research is broadening to overcome these difficulties.

Compared to the metamaterial UWB antenna [30], the new design antenna provides better gains over the operating bands and improved efficiency, although the antenna dimensions are the same. Dielectric material is applied in the new design with lower loss, which simplifies the antenna fabrication process. Sharp current flow, radiation with low cross polarization and high correlation factors are achieved from the new design, which enables it to be used as a microwave imaging sensor. 

A planar-patterned metamaterial concept was used in [31,32]. A coupled capacitive-inductive circuit is formed using the patterned patch and the ground plane. The dimensions of the antenna in [31] were 28 mm × 32 mm and the bandwidth covered the range of 5.3–8.5 GHz, with the gain above 4 dBi. The dimensions of the antenna in [32] were 27.6 mm × 31.8 mm, and the bandwidth covered the range of 3.85–15.62 GHz, with the average gain of 5.42 dBi. Our antenna design is better than that of [31,32] with respect to antenna size, impedance bandwidth and gain. A metamaterial unit cell antenna has been proposed for UWB applications [33]; however, this antenna size is large, has lower directivity, has lower gain and does not cover the UWB range (3.1–10.6 GHz). 

An elliptical tapered slot antenna has been described for UWB medical imaging [34], whose electrical dimensions were 0.52 λ × 0.52 λ at a lower frequency of 3.10 GHz. However, the antenna dimensions (50 mm × 50 mm) were too large. A metamaterial antenna was investigated for UWB application with a modified SRR and capacitive loaded strip (CLS), where the electrical dimensions were 0.21 λ × 0.20 λ at a lower frequency of 2.90 GHz [35]. This antenna covered the frequency range of 2.9–9.9 GHz. However, the UWB band (3.1–10.6 GHz) was not completely covered. A microstrip-fed “Dark Eyes” antenna was studied for near-field microwave sensing [10]. The overall dimensions were 22.25 mm × 20 mm. The gain was not reported. A UWB antenna with a negative index metamaterial was reported [36]. The antenna’s overall size was 16 mm × 21 mm, and its gain was 1.0–5.16 dBi. The antenna covered the frequency band from 3.40 to 12.5 GHz, with a fractional bandwidth 114.50%; the UWB band (3.1–10.6 GHz) was not covered completely. Several ultra-wideband antennas were presented with low distortion, compact size, and different shapes for microwave imaging [37,38,39]. Each antenna had its own advantages and disadvantages. Some of these antenna had low radiation and/or lower gain and lacked of a planar structure.

This paper introduces a microwave imaging sensor based on a novel compact metamaterial UWB antenna using a new technique. This metamaterial antenna is based on four unit cells of metamaterial on the patch with a partial ground while maintaining an impedance bandwidth from 3.05 GHz to 15 GHz. Metamaterial unit cells (a combination of a modified SRR and a CLS) simultaneously show both negative permittivity and negative permeability. The parametric analysis is performed to achieve the optimal results. A high correlation factor can be found between the transmitting antenna and the virtual probes in both the E-plane and the H-plane, which enables this metamaterial antenna to be used as microwave imaging sensor. A combination of theory and experimental techniques such as modified SRR, CLS and wire with three slots in the partial ground plane have been applied in this paper, which bears the novelty of the proposed metamaterial antenna as microwave imaging sensor. This proposed metamaterial antenna is very much suitable for medical instrumentation industry.

## 2. Unit Cell Design Architecture

**Figure 1:**
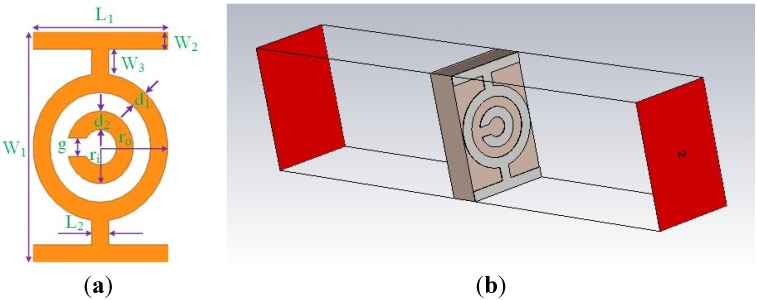
(**a**) The front side of the unit cell and (**b**) the simulation geometry.

A metamaterial unit cell is used to initiate the proposed antenna design architecture. The goal is to attain a unit cell design having a resonance characteristic in the frequency range of 3.1 GHz to 10.6 GHz. Various reputed methods are used for metamaterial structure design, such as SRRs [16,17]. In this research, the initial unit cell is based on an SRR structure. The SRR is made of two loops: *i.e.*, a smaller loop within a bigger one, with slots incorporated onto each loop at opposite ends [16]. A perpendicular magnetic field reacts with a magnetically resonant structure such as an SRR, which can be used to create negative permeability. Gaps (splits) added to the ring, introduce capacitance, which allows for the control of the resonant characteristic of the structure. The first unit cell is the modified rectangular SRR shown in Figure 1. The modification is the closing of the loop on the outer ring, which reduces the series capacitance of the SRR. Furthermore, closing the outer ring enhances the coupling between the outer and inner rings, which enables a wide backward-wave passband [40]. The unit cell is printed onto a FR4 substrate with a dielectric constant of 4.6, and a thickness of 1.6 mm. Two CLSs are assembled to the modified SRR unit cell to achieve a resonance within 3.1–10.6 GHz. The CLSs which act as electric dipoles are I-shaped striplines that mimic long metallic wires [41]. The combined structure (modified SRR and CLS) allows for simultaneous electric and magnetic resonance because the SRR resonates with a perpendicular magnetic field and the capacitive loaded strip (which is basically an electric dipole) resonates through a parallel electric field [42]. The two resonance mechanism enables a lower resonance through the united induced current for the total design. The unit cell design specifications are summarized in Table 1.

**Table 1:** The design parameters for the unit cell.

Parameter	Dimension (mm)	Parameter	Dimension (mm)
*W*_1_	6.6	*d*_1_	0.5
*W*_2_	0.528	*d*_2_	0.5
*W*_3_	0.703	*g*	0.5
*L*_1_	3.872	*r*_i_	1
*L*_2_	0.484	*r*_o_	2

The metamaterial (MTM) unit cell was simulated using Computer Simulation Technology (CST) software based on the finite-difference time domain (FDTD) approach for attaining the S-parameters. The unit cell simulation geometry is shown in Figure 1b. The structure used for testing was located between two waveguide ports situated on each side of the *x*-axis. An electromagnetic wave was excited along the *x*-axis. A perfectly-conducting electrical boundary condition was applied along the walls perpendicular to the *y* axis, and a perfectly-conducting magnetic boundary was applied at the walls perpendicular to *z*-axis. A frequency domain solver is applied to simulate this metamaterial structure. The normalized impedance is matched to 50 Ω. This simulation is executed over the 3–15 GHz frequency range.

**Figure 2:**
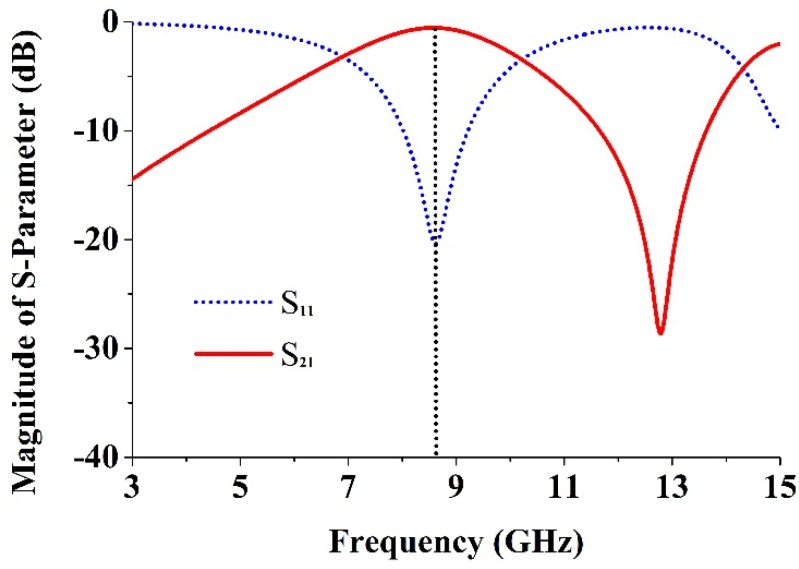
The magnitude of the *S*-parameters (*S*_11_ and *S*_21_) for the reported unit cell (Figure 1).

The *S* parameters (the reflection coefficient, *S*_11_, and the transmission coefficient, *S*_21_) were obtained through simulation and entered into the Math CAD software. A transmission peak corresponding to a left-handed band that occurs at 8.6 GHz is shown in Figure 2. From the self-resonance, the overlap, and the larger overall current responses with respect to existing SRRs designs, it is clear that the proposed metamaterial’s magnetic response is the main advantage. The Nicolson-Ross-Weir approach [19,30] was used to extract the constitutive effective parameters from *S*_21_ and *S*_11_, including the refractive index *n*_r_, the relative effective permittivity ε_r_, and the permeability μ_r_. These following equations are achieved individually in accordance with:

(1) $${\text{ε}}_{\text{r}}=\frac{2}{jk_{0}d}*\frac{1-V_{1}}{1+V_{1}}$$

(2) $${\text{μ}}_{\text{r}}=\frac{2}{jk_{0}d}*\frac{1-V_{2}}{1+V_{2}}$$

(3) $$n_{\text{r}}=\sqrt{{\text{ε}}_{\text{r}}{\text{μ}}_{\text{r}}}$$

(4) $$V_{1}=S_{21}+S_{11}$$

(5) $$V_{2}=S_{21}-S_{11}$$

where: *k*_0_ = ω/*c* ; ω = 2π*f*, angular frequency; *d* = Slab thickness and *c* = Speed of light.

**Figure 3:**
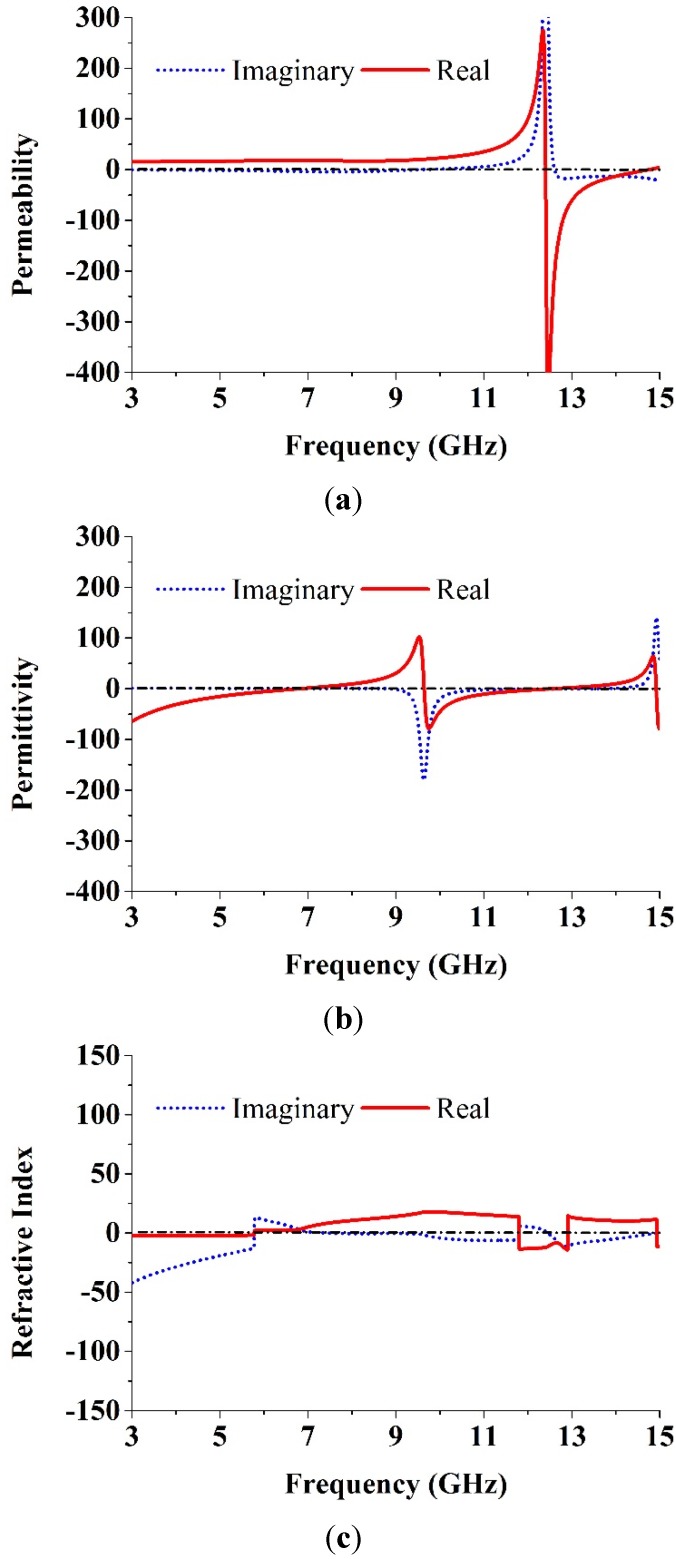
The proposed unit cell (**a**) permeability, (**b**) permittivity and (**c**) refractive index.

The effective parameters are retrieved using Equations (1)–(5). The refractive index, the permeability, and the permittivity of the unit cell are plotted in Figure 3. The negative frequency regions are listed in Table 2. From Table 2, the MTM unit cell is found to belong to a different resonant property in the frequency zone of negative value. The parameters of the proposed MTM design are significantly improved compared with those of previously reported MTMs that also possess negative values over a broad band [20,22,23,27,29,30,33,36].

**Table 2:** The permeability, permittivity and refractive index in the negative frequency zone.

Parameter	Negative Frequency Zone (GHz)
Permeability, µ_r_	12.40–14.75
Permittivity, ɛ_r_	3–6.82, 9.65–12.56, 14.93–15
Refractive index, *n*_r_	3–5.77, 11.81–12.90, 14.94–15

## 3. Antenna Design and Fabrication

**Figure 4:**
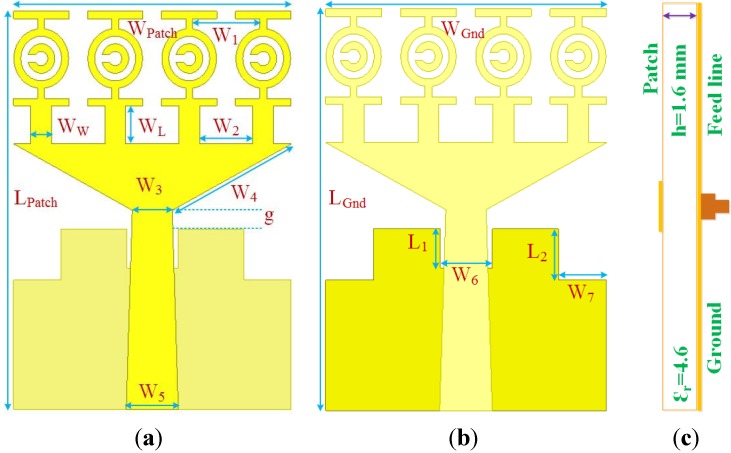
The reported MTM antenna (**a**) front view, (**b**) back view and (**c**) cross-sectional view.

The proposed antenna design architecture (front view, bottom view and cross-sectional view) is shown in Figure 4. The antenna consists of four metamaterial unit cells along one axis on the patch and a partial ground fed by a microstrip trident-shaped strip. The antenna is printed on FR4 material with a dielectric constant of 4.6 and 1.6 mm thickness. The overall antenna dimensions are 19.36 mm × 27.72 mm × 1.6 mm, where the electrical dimensions are 0.20 λ × 0.29 λ × 0.017 λ at the lower frequency band of 3.1 GHz. The MTM unit cells are homogeneous to each other. A Sub Miniature Version A connector is attached to the port that delivers a 50 Ω impedance. The optimal design parameters are summarized in Table 3.

**Table 3:** The antenna design parameters according to Figure 4.

Parameter	Dimension (mm)	Parameter	Dimension (mm)
*W*_Patch_	19.36	*W*_6_	3.575
*L*_Patch_	27.72	*W*_7_	3.3
*g*	1.32	*W*_L_	2.64
*W*_1_	4.676	*W*_W_	1.452
*W*_2_	3.708	*W*_Gnd_	19.36
*W*_3_	2.78	*L*_Gnd_	27.72
*W*_4_	9.51	*L*_1_	2.695
*W*_5_	3.63	*L*_2_	3.52

**Figure 5:**
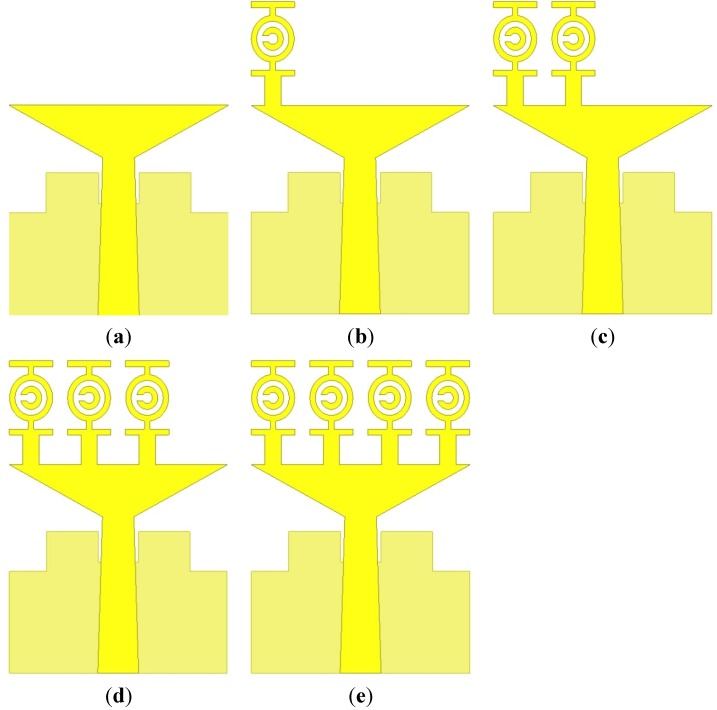
The proposed antenna. (**a**) No unit cell, (**b**) One unit cell, (**c**) Two unit cells, (**d**) Three unit cells and (**e**) Four unit cells (proposed).

**Figure 6:**
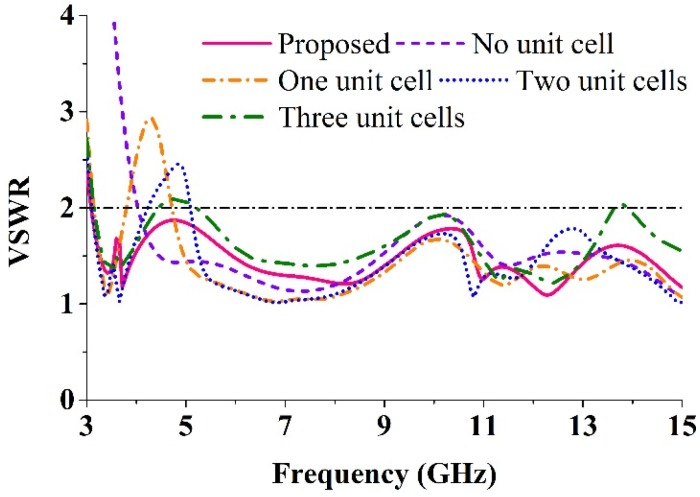
The effects of the radiating patch unit cell on the VSWR.

**Figure 7:**
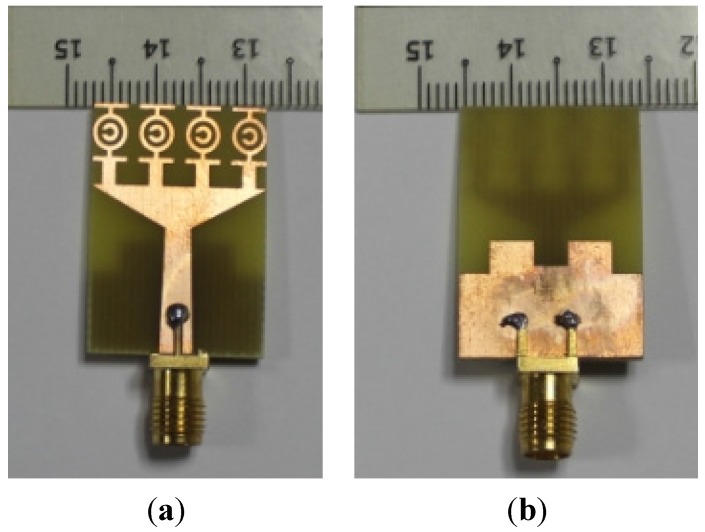
Photograph of the proposed UWB antenna (**a**) top view and (**b**) back view.

Figure 5 illustrates the evolution of the reported antenna. The effects of the radiating patch unit cell on the VSWR are shown in Figure 6. Table 4 shows the comparisons of the effects of the unit cell on the VSWR, which can be found in Figure 6. The antenna with no unit cell achieves a frequency range of 4.02–15 GHz. However, this antenna does not cover the entire UWB range (3.1–10.6 GHz) approved by the Federal Communications Commission. The use of a unit cell, attempts to shift the lower frequency to 3 GHz. A proper analysis was performed to support the usage of four unit cells instead of another number, which is clarified in Figure 6. Apparently, the proposed antenna design with four unit cells provides the optimal computed results regarding VSWR while covering the standard UWB frequency range (3.1–10.6 GHz). The fabricated photograph of the antenna is shown in Figure 7. 

**Table 4:** Comparisons of the unit cell effects on the VSWR according to Figure 5.

Number of Unit Cell	Covering Frequency Region (VSWR < 2), GHz
No unit cell	4.02–15
One unit cell	3.18–3.78, 4.74–15
Two unit cell	3.12–4.2, 5.1–15
Three unit cell	3.18–4.44, 5.22–13.62, 13.92–15
Four unit cell (Proposed)	3.03–15

## 4. Antenna Performance

**Figure 8:**
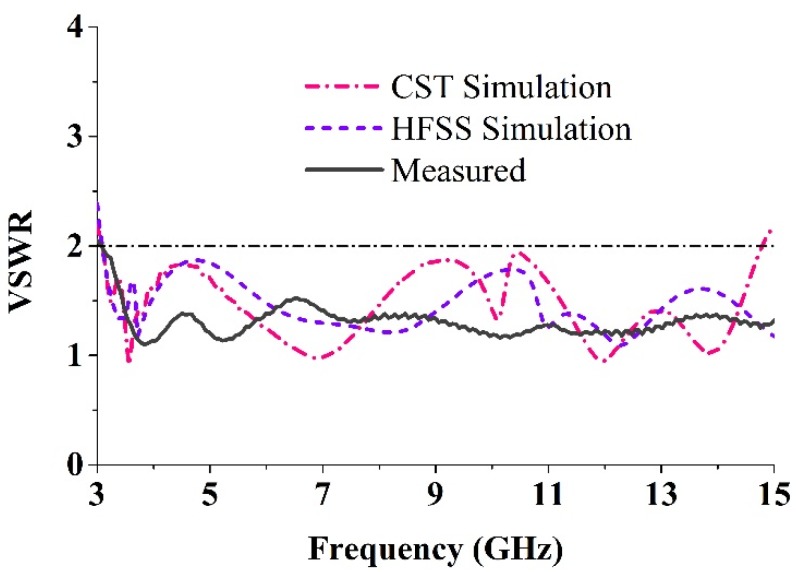
Measured and simulated VSWR curves.

The simulations were performed using the HFSS and CST Microwave Studio to achieve VSWR < 2 over the entire ultra-wideband spectrum. Measurements are performed using a power network analyzer (N5227A) that covers the range of 10 MHz–67 GHz. Figure 8 shows the VSWR of the simulation and the measurements. From the HFSS simulation, an impedance bandwidth covering 3.03–15 GHz is achieved while a range of 3.02–15 GHz is obtained from the CST simulation. The measured impedance bandwidth is 3.05–15 GHz which is in a good agreement with the simulations. 

**Figure 9:**
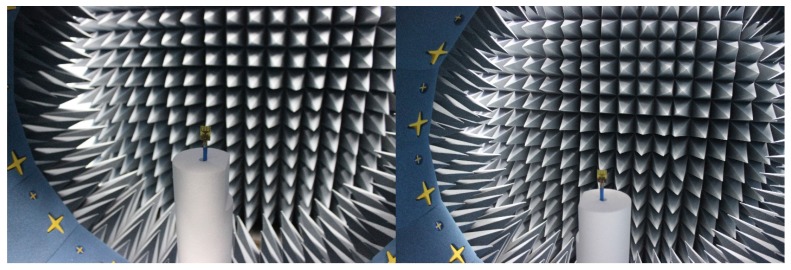
The antenna measurement setup in the UKM StarLab.

**Figure 10:**
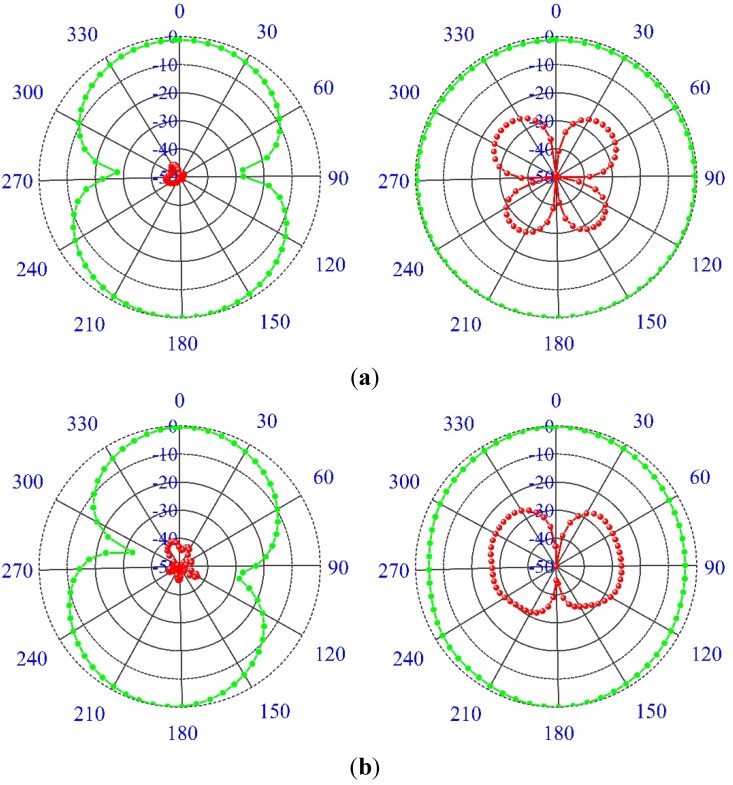

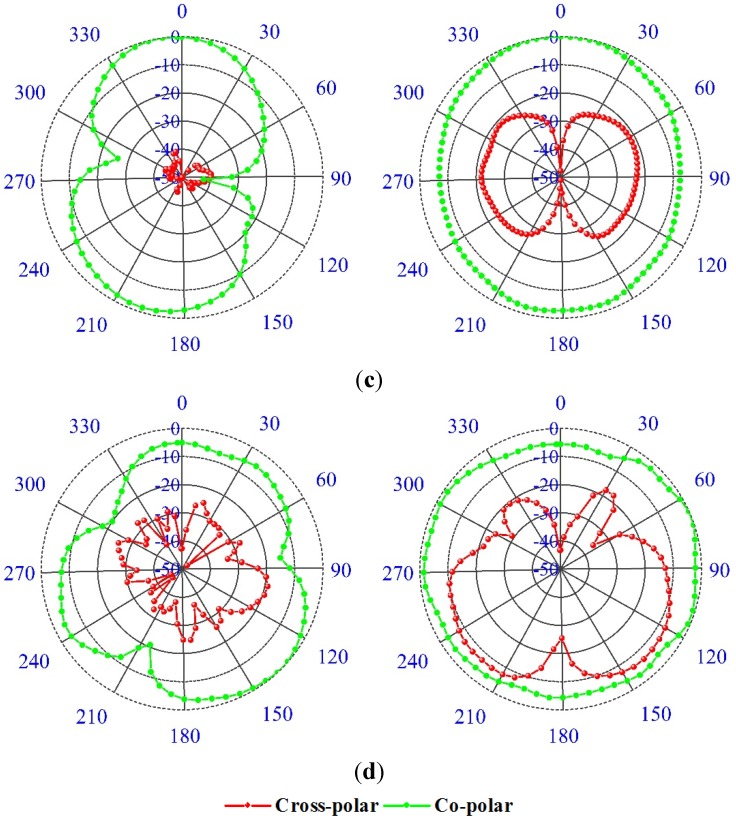
The measured radiation pattern at (**a**) 3.5 GHz, (**b**) 5.5 GHz, (**c**) 7.5 GHz and (**d**) 10.5 GHz.

The spherical scanning system was used in the near-field antenna measurement system as shown in Figure 9. The measured radiation pattern is illustrated in Figure 10 for (a) 3.5; (b) 5.5; (c) 7.5; and (d) 10.5 GHz at the E-plane and the H-plane. Two-dimensional (2D) radiation patterns are shown to indicate cross and co-polarization. To denote the co-polarization and cross-polarization, *E*_θ_ and *E*_φ_ are plotted, respectively, where the *x*-*z* plane is considered as the H-plane and the *y*-*z* plane is considered as the E-plane. The antenna is found to be nearly omni-directional in the H-plane and bi-directional in the E-plane. In addition, the cross-polarization was lower than the co-polarization.

**Figure 11:**
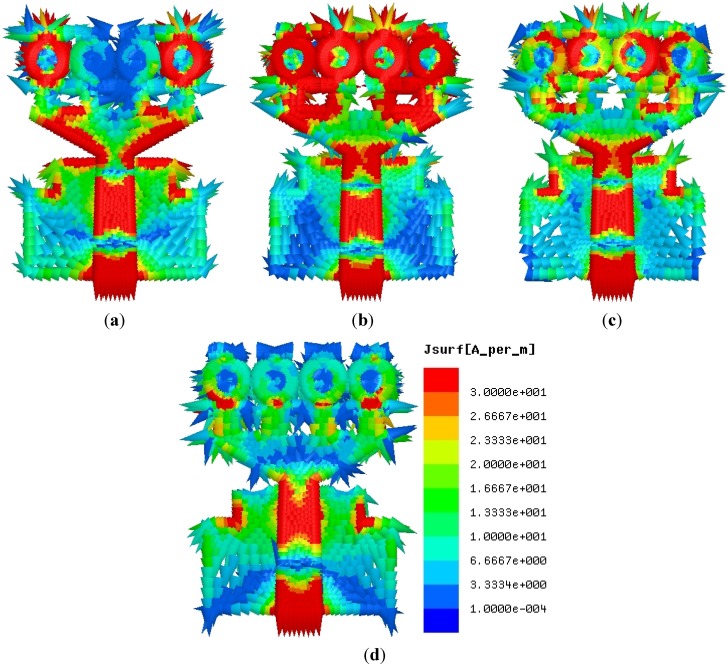
The surface current distribution at. (**a**) 3.5 GHz, (**b**) 5.5 GHz, (**c**) 7.5 GHz and (**d**) 10.5 GHz.

The surface current distribution at frequencies of 3.5, 5.5, 7.5 and 10.5 GHz, are shown in Figure 11. The current flow is observed to be dominants around the unit cells on the patch with the transmission line, *i.e.*, these unit cells play important roles for originating the resonances and attaining the UWB frequency bands. The 1st and 4th unit cells at 3.5 GHz, and the, 1st, 2nd, 3rd, and 4th unit cells at 5.5 GHz are affected by the current flow. At 7.5 GHz and 10.5 GHz, the unit cells are affected partially by the current flow. This response ensures that the performance of this UWB antenna depends on the unit cells on the patch and the feeding to create an ultra-wide frequency band. However, it is observed that the surface current conducts a sharp flow at both the slotted ground plane and the radiating patch with metamaterial structures and microstrip transmission lines.

**Figure 12:**
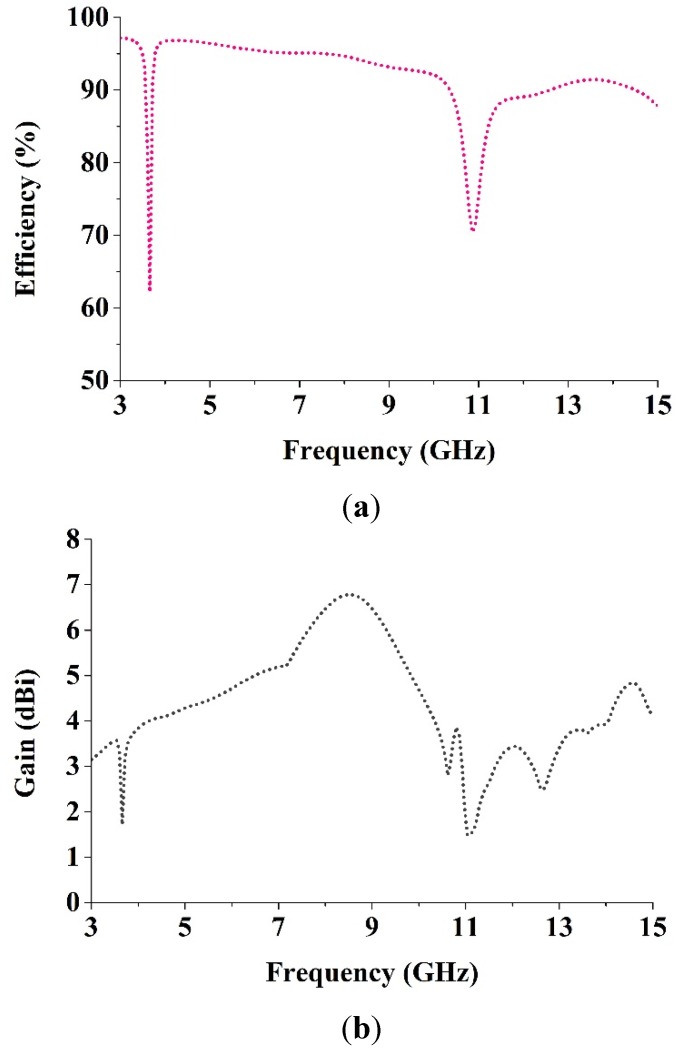
(**a**) The radiation efficiency and (**b**) the measured gain of the antenna.

Figure 12a shows that the average radiation efficiency is 92.02% where the maximum and minimum efficiency are 97.15% and 62.14%, respectively, which are acceptable and better than those of existing antennas [30,36]. Figure 12b shows that the proposed antenna has average gain 4.38 dBi, with the maximum and minimum gains of 6.78 dBi and 1.48 dBi, respectively, which are better than newly proposed metamaterial antennas in [30,36].

Table 5 contains the comparisons between the proposed UWB antenna and the existing antennas. The proposed antenna and the existing antennas [10,30,31,32,33,34,35,36,37,38,39] were also studied to ensure an impartial comparison, where all the reference antennas reported in the literature review cover the ultra-wideband spectrum. The performance parameters, such as applications, 10-dB bandwidth, dimensions, electrical dimensions, fractional bandwidth and gain were discussed. Although the proposed antenna may not have a better gain than those of the references [31,32,34,37], a good fractional bandwidth (FBW, 132.41%) with a smaller electrical dimension was achieved. Therefore, the proposed UWB metamaterial antenna can offer good compact characteristics while maintaining much smaller dimensions than the designs in [31,32,34,37,38,39]. 

**Table 5:** Comparisons between the proposed ultra-wideband (UWB) antenna and the existing antennas.

Antennas	Application	BW GHz (−10 dB)	Dimension (mm^2^)	Electrical Dimension	FBW (%)	Gain dBi
[10]	Microwave Sensing	2.70–9.70	22.25 × 20	0.20 λ × 0.18 λ	112.90	not reported
[30]	Microwave Sensing	3.10–15.00	19.36 × 27.72	0.20 λ × 0.29 λ	131.50	1.2~6.57
[31]	Ultra-Wideband	5.30–8.50	28 × 32	0.50 λ × 0.57 λ	46.38	1~7.2
[32]	Ultra-Wideband	3.85–15.62	27.6 × 31.8	0.36 λ × 0.41 λ	120.91	0.5~8.36
[33]	Ultra-Wideband	5.20–13.90	25 × 25	0.43 λ × 0.43 λ	91.01	1.2~3.85
[34]	Medical Imaging	3.10–11.00	50 × 50	0.52 λ × 0.52 λ	112.01	4.3~10.8
[35]	Ultra-Wideband	2.90–9.90	22 × 21	0.21 λ × 0.20 λ	109.38	−1.0~5.0
[36]	Microwave Imaging	3.40–12.50	16 × 21	0.18 λ × 0.24 λ	114.50	1.0~5.16
[37]	Microwave Imaging	1.15–4.40	75 × 75	0.29 λ × 0.29 λ	117.12	2.0~8.0
[38]	Microwave Imaging	3.80–11.85	30 × 30	0.38 λ × 0.38 λ	102.00	not reported
[39]	Microwave Imaging	4.00–9.00	30 × 30	0.40 λ × 0.40 λ	76.92	2.0~6.0
Proposed	Microwave Sensing	3.05–15.00	19.36 × 27.72	0.20 λ × 0.28 λ	132.41	1.48~6.78

## 5. Time Domain Performance

The time domain properties of the antenna are calculated using the full-wave simulation software CST Microwave Studio. By the use of virtual probes located a distance of 500 mm from the feeding point of the monopole antennas, the corresponding received signals *r*(*t*) could be readily obtained. Thus, we could calculate the correlation between the time-domain input pulse signal *s*(*t*) and the received signals *r*(*t*) observed by these probes to evaluate the signal preserving capabilities of these antennas. Having an improved level of correlation between the received and transmitted signals is essential in UWB impulse radio communications for avoiding modulated information loss. The definition of the correlation factor is given by:

(6) $$F=\max_{\text{τ}}|\frac{\int_{-\infty }^{+\infty }s(t)r(t-\text{τ})}{\sqrt{\int_{-\infty }^{+\infty }s{\left(t\right)}^{2}\text{d}t.\int_{-\infty }^{+\infty }r{\left(t\right)}^{2}\text{d}t}}|$$

where τ is the delay that is varied to make *F* in Equation (6) a maximum. To alleviate the signal distortions caused by the bandwidth mismatch between the antenna and the input source pulse, a UWB signal introduced in [30,36] is assumed to excite these antennas. This UWB signal is the 5th-derivative of the Gaussian pulse and is given by:

(7) $$s\left(t\right)=GM_{5}\left(t\right)=C\left(-\frac{t^{5}}{\sqrt{2\text{π}}{\text{σ}}^{11}}+\frac{10t^{3}}{\sqrt{2\text{π}}{\text{σ}}^{9}}-\frac{15t}{\sqrt{2\text{π}}{\text{σ}}^{7}}\right)\times \exp\left(-\frac{t^{2}}{2{\text{σ}}^{2}}\right)$$

**Figure 13:**
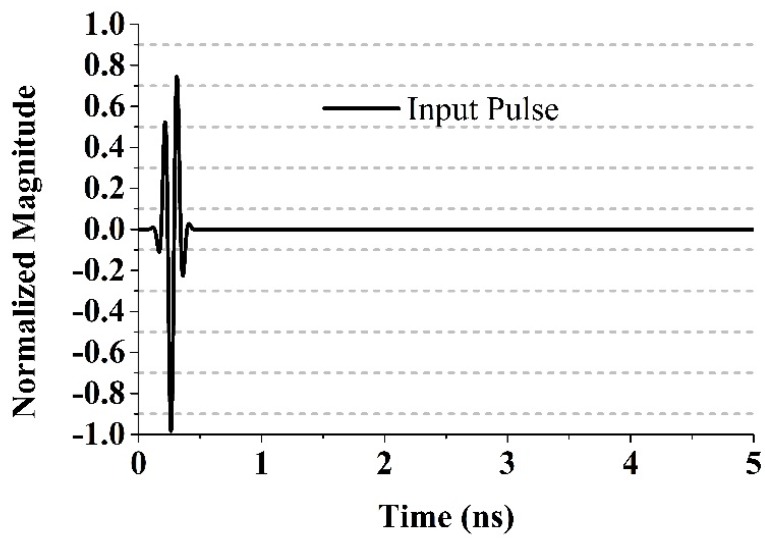
The input pulse signal in the time domain.

*C* is a constant that can be chosen to comply with peak power spectral density suggested by the FCC, and σ must be 51 ps to ensure that the shape of the spectrum complies with the FCC spectral mask. The shape of its spectrum is determined through the envelope of this pulse. The 5th derivative of the Gaussian pulse has been generated, when the Gaussian pulse is differentiated and generated five times. Figure 13 illustrates this pulse signal in time domain. The impedance bandwidth (VSWR < 2) of the input signal is found to be from 3.05 GHz to 15 GHz, which lies in the impedance bandwidth of the UWB antenna.

**Figure 14:**
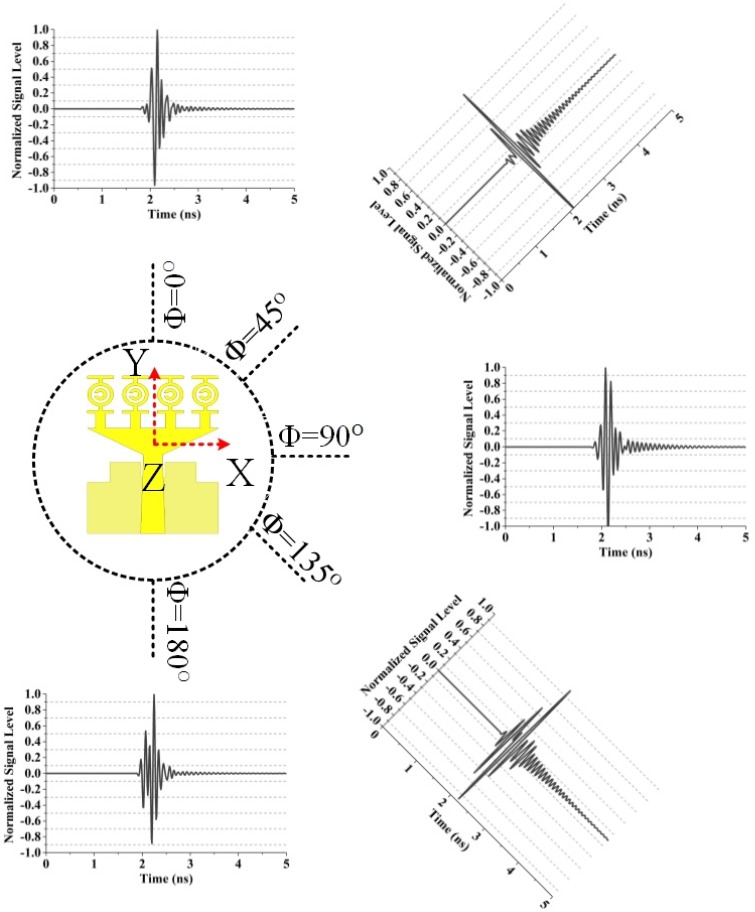
Normalized received signals by the virtual probe for θ = 90° and varying φ in the E-plane.

**Figure 15:**
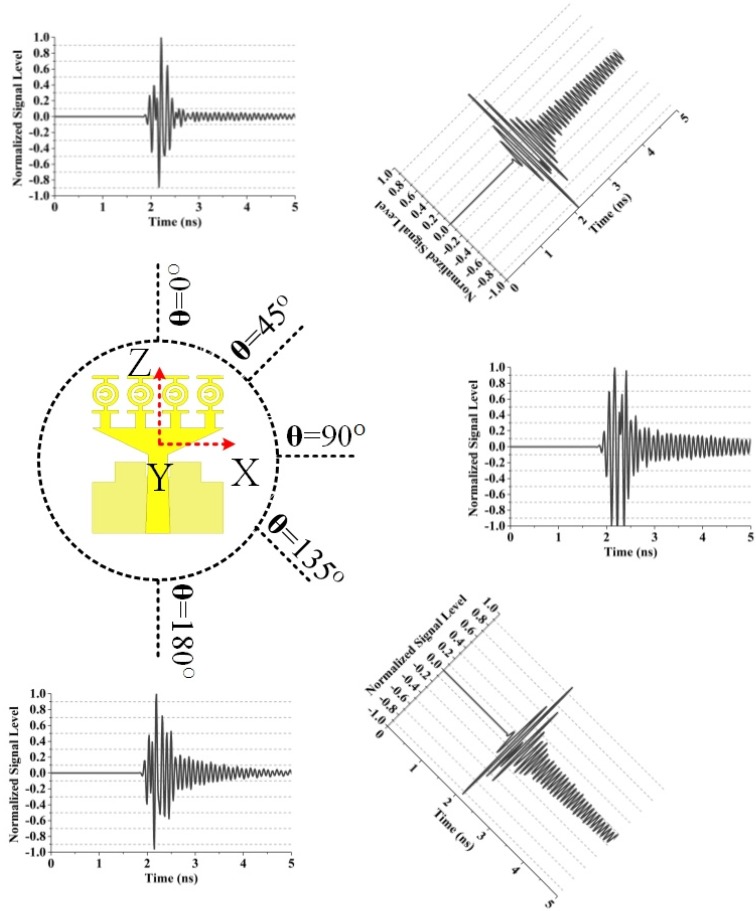
Normalized received signals by the virtual probe for φ = 0° and varying θ in the H-plane.

The distance was assumed to be 500 mm between the transmitting and the virtual probes in both the E-plane and the H-plane. Figure 14 illustrates the normalized received signals by the virtual probe for θ = 90° and varying φ in the E-plane. The normalized received signals by the virtual probe for φ = 0° and varying θ in the H-plane is shown in Figure 15. The correlation factors of the proposed UWB antenna and the received signals of the virtual probes are summarized in Table 6 (E-plane) and Table 7 (H-plane), respectively. We have seen from the Figure 14 that the correlation factor is the highest (0.87) when Φ = 90° and the lowest (0.83) when Φ = 180° (Table 6). In the H-plane, the highest correlation factor (0.89) is found when θ = 90° and the lowest (0.85) when θ = 180°, which is observed from the Figure 15 (Table 7). The correlation factor (H-plane) is more stable than that of E-plane. The distortions of the received signals were slightly alleviated compared with the results in [30], and severe ringing and spreading of the received signals could be observed, which are similar to the results in [30]. The transmitted signals with severe ringing and pulse-width spreading may be caused by the energy storage effects of the dielectric substrate when the prototype antenna was transmitting pulse signals.

**Figure 16:**
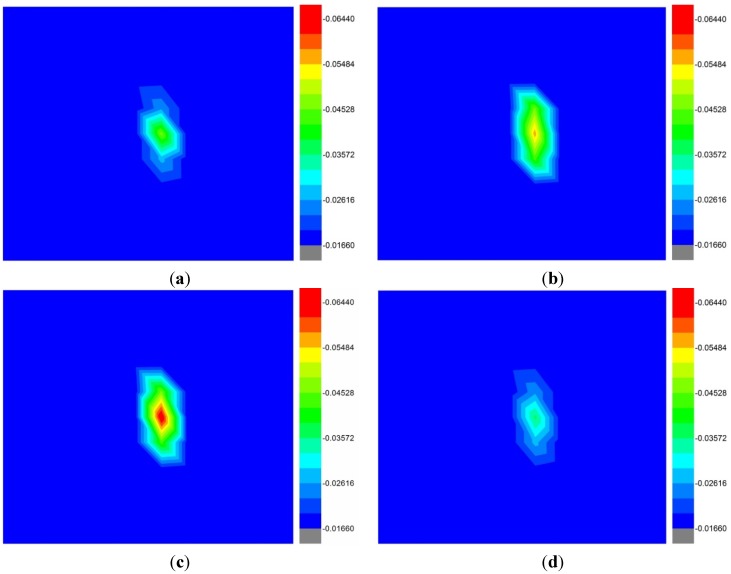
The acquired images from 2-D scanning at (**a**) 3.5 GHz, (**b**) 5.5 GHz, (**c**) 7.5 GHz and (**d**) 10.5 GHz.

The antenna was tested to verify the capability of the antenna as a microwave imaging sensor used in an aperture scanning method [4,30] to detect tumors in a breast phantom (lossy dielectric). The breast phantom of 140 mm × 140 mm dimension is applied for the test, with one spherical shape tumor simulant with a radius 6. The permittivity and conductivity of the tumor simulant were 67 and 5 S/m, respectively. The guidelines of [4,30] are followed for the simulation and the scanning method. The transmission S-parameter (*S*_21_) was obtained in an area of 120 mm × 120 mm with a 5 mm spatial sampling rate. The images (Figure 16) were obtained at 3.5 GHz, 5.5 GHz, 7.5 GHz, and 10.5 GHz. The tumor simulant was easily detected at 7.5 GHz compared to other frequency 3.5 GHz, 5.5 GHz, and 10.5 GHz, but the tumor shape was not clearly identifiable because it was electrically too small.

**Table 6:** Correlation factors of the proposed UWB antenna for the virtual probes in the E-plane according to Figure 14.

Probe Position		Correlation Factor
θ = 90°	Φ = 0°	0.84
θ = 90°	Φ = 45°	0.85
θ = 90°	Φ = 90°	0.87
θ = 90°	Φ = 135°	0.86
θ = 90°	Φ = 180°	0.83

**Table 7:** Correlation factors of the proposed UWB antenna for the virtual probes in the H-plane according to Figure 15.

Probe Position		Correlation Factor
θ = 0°	Φ = 0°	0.86
θ = 45°	Φ = 0°	0.87
θ = 90°	Φ = 0°	0.89
θ = 135°	Φ = 0°	0.88
θ = 180°	Φ = 0°	0.85

## 6. Conclusions

In this paper, a compact metamaterial UWB antenna has been presented as a microwave imaging sensor with a high correlation factor. This microwave antenna sensor consists of four unit cells along one axis and each unit cell discloses negative permittivity, negative permeability, and negative refractive index simultaneously. The overall antenna size is 19.36 mm × 27.72 mm × 1.6 mm where the electrical dimension is 0.20 λ × 0.28 λ × 0.016 λ at the 3.05 GHz lower frequency band. It provides a fractional bandwidth (132.41%) covering the working frequency range 3.05–15 GHz (VSWR < 2), a maximum radiation efficiency of 97.15% and maximum gain of 6.78 dBi. The performance of the proposed antenna was tested using an aperture scanning method to detect a tumor in a lossy dielectric breast phantom. The obtained high correlation factor verifies that the metamaterial antenna has the capability to detect tumour simulants as an imaging sensor. A high correlation factor, tumour detecting capability, and stable gain with efficiency ensures the potentiality of using it as an imaging sensor.
